# Congenital coronary artery-to-pulmonary fistula with giant aneurysmal dilatation and thrombus formation: a case report and review of literature

**DOI:** 10.1186/s12872-021-02077-4

**Published:** 2021-06-04

**Authors:** Xuanqi An, Shaoxian Guo, Huawei Dong, Yida Tang, Lin Li, Xuejing Duan, Shaodong Ye

**Affiliations:** 1 State Key Laboratory of Cardiovascular Disease, Center of Internal Medicine, Fuwai Hospital, National Center for Cardiovascular Diseases, Chinese Academy of Medical Sciences and Peking Union Medical College, No. 167 Beilishi Rd, Xicheng District, Beijing, 100037 People’s Republic of China; 2 State Key Laboratory of Cardiovascular Disease, Center of Surgery, National Center for Cardiovascular Diseases, Fuwai Hospital, Chinese Academy of Medical Sciences and Peking Union Medical College, Beijing, 100037 People’s Republic of China; 3 State Key Laboratory of Cardiovascular Disease, Department of Pathology, Fuwai Hospital, National Center for Cardiovascular Diseases, Chinese Academy of Medical Sciences and Peking Union Medical College, Beijing, 100037 People’s Republic of China

**Keywords:** Coronary artery-to-pulmonary artery fistula, Giant coronary aneurysm, Thrombus, Coronary angiography, Surgery ligation

## Abstract

**Background:**

Coronary artery-to-pulmonary artery fistula is a rare disorder characterized by abnormal vascular communication between the coronary artery and pulmonary artery. While most patients remain asymptomatic, some might exhibit symptoms of myocardial ischemia, congestive heart failure, or even sudden cardiac death if coronary aneurysm, thrombosis, infective carditis, or other congenital cardiac defects coexist.

Case presentation

We present a 66-year-old male complaining of angina pectoris with a history of hypertension and active smoking. He was diagnosed with a coronary aneurysm based on coronary computed tomography angiography. We subsequently identified a coronary artery-to-pulmonary artery fistula with giant aneurysmal dilation on coronary angiography. Ultimately we conducted surgery ligation and aneurysmorrhaphy. During surgery, we discovered newly formed thrombus within the aneurysmal cavity. Histological analysis of the aneurysmal wall supported the diagnosis of the congenital disorder. Our patient was successfully discharged and remained asymptomatic at two months of follow-up.

**Conclusion:**

We presented a rare and complex combination of congenital coronary artery-to pulmonary artery fistula, giant coronary aneurysmal dilatation, and thrombosis through multi-modality evaluations.

**Supplementary Information:**

The online version contains supplementary material available at 10.1186/s12872-021-02077-4.

## Background

Coronary artery-to-pulmonary artery fistula (CPAF) is a rare coronary artery anomaly defined as the abnormal communication between the coronary artery and pulmonary artery [1]. It mostly remains asymptomatic before incidentally discovered by coronary computed tomography angiography (CCTA) [2]. However, it could also manifest as angina pectoris, congestive heart failure, pulmonary hypertension, or even sudden cardiac death depending on the severity of the left–right shunt and whether a concomitant ruptured aneurysm exists [2]. The treatment regimen for CPAF, including oral antiplatelet or anticoagulant medications, interventional endovascular procedures, and ultimately, surgical ligation, should be individually tailored [3]. Here we report a rare case of CPAF with coronary aneurysmal formation and concomitant thrombosis.

## Case Presentation

**Fig. 1:**
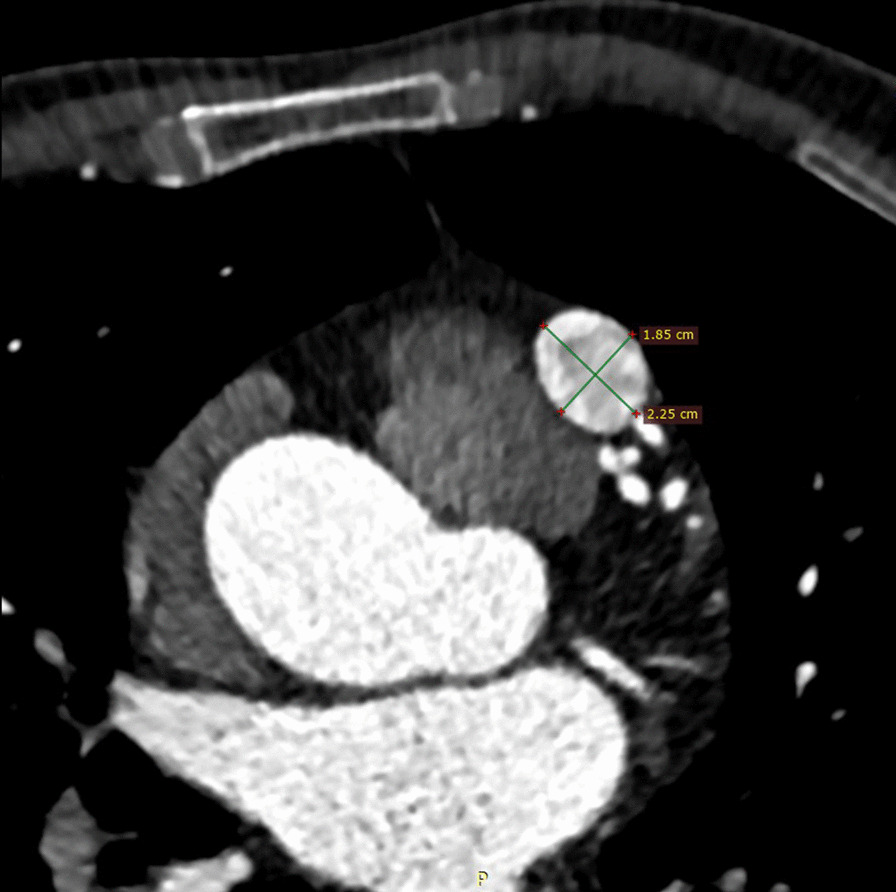
Coronary computed tomography angiogram in the local hospital spotted a coronary aneurysm of 2.23 * 1.81 cm

**Fig. 2:**
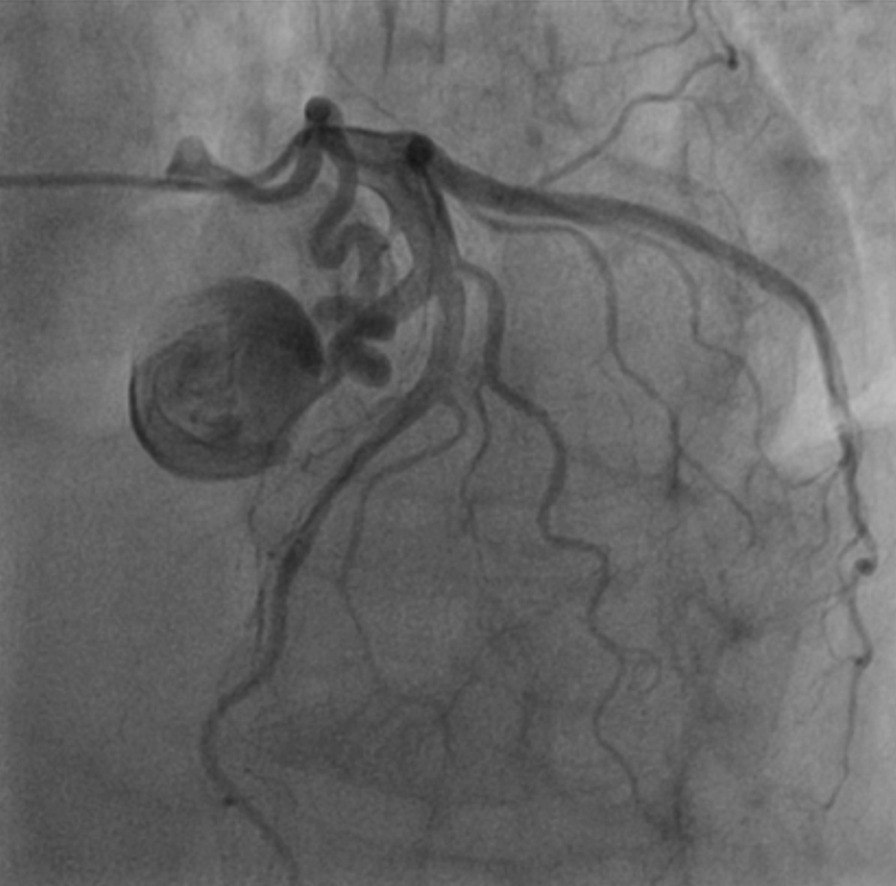
Coronary angiography identified a coronary artery to pulmonary artery fistula with giant aneurysmal dilation through Left Anterior Oblique angulation

**Fig. 3:**
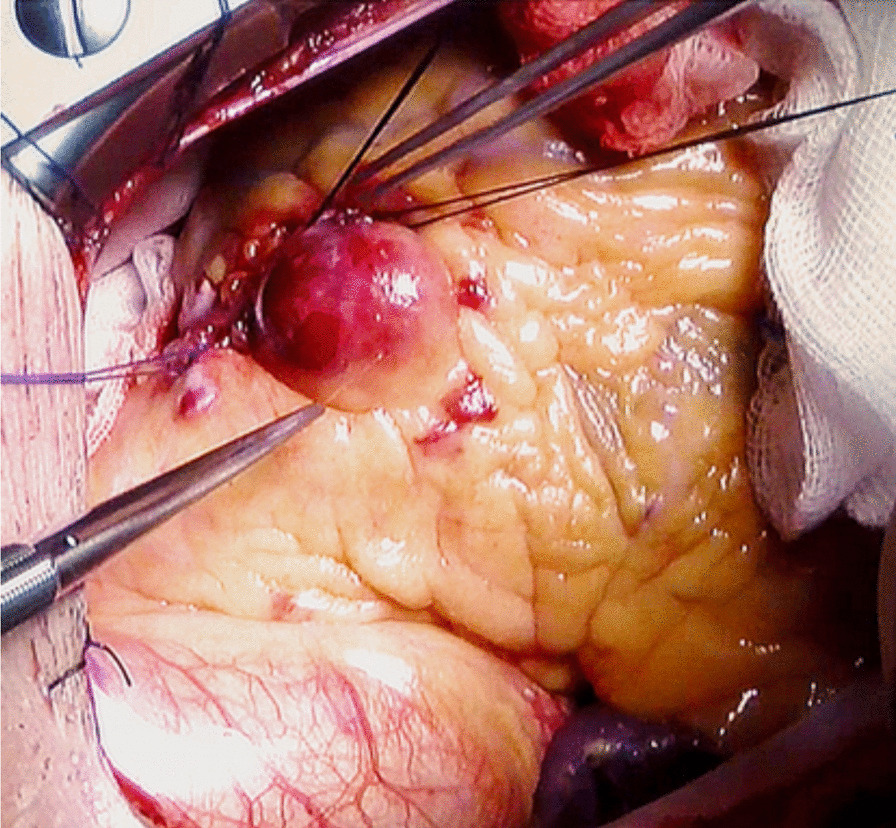
We spotted the coronary aneurysm of 3 cm in diameter located within the epicardial adipose tissue during surgery

**Fig. 4:**
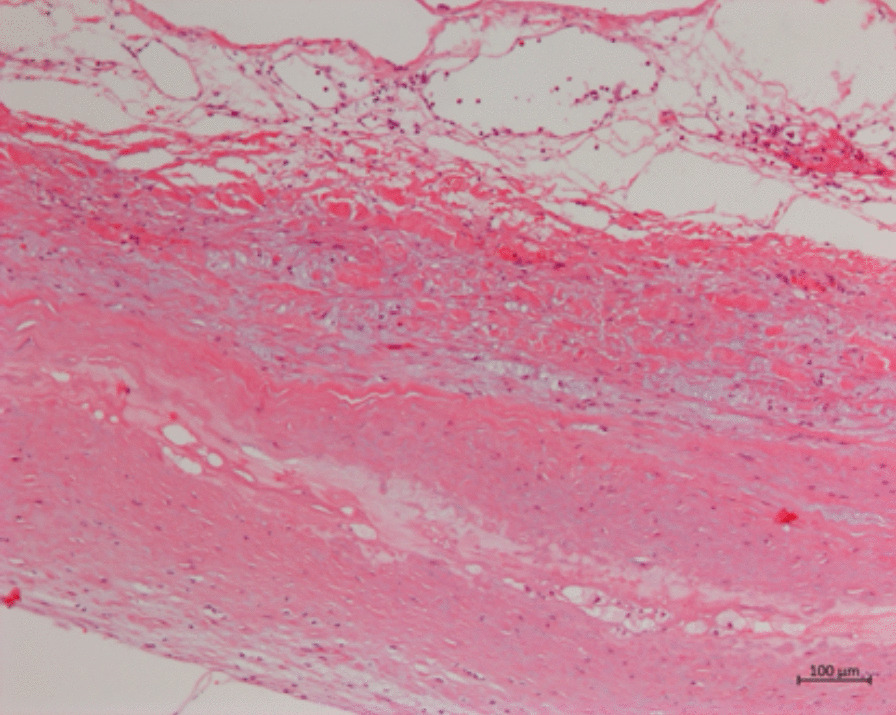
Tissue biopsy of the coronary aneurysmal wall by hematoxylin–eosin staining showed moderate intimal fibrosis, degenerated media, and decreased smooth muscle layer

**Fig. 5:**
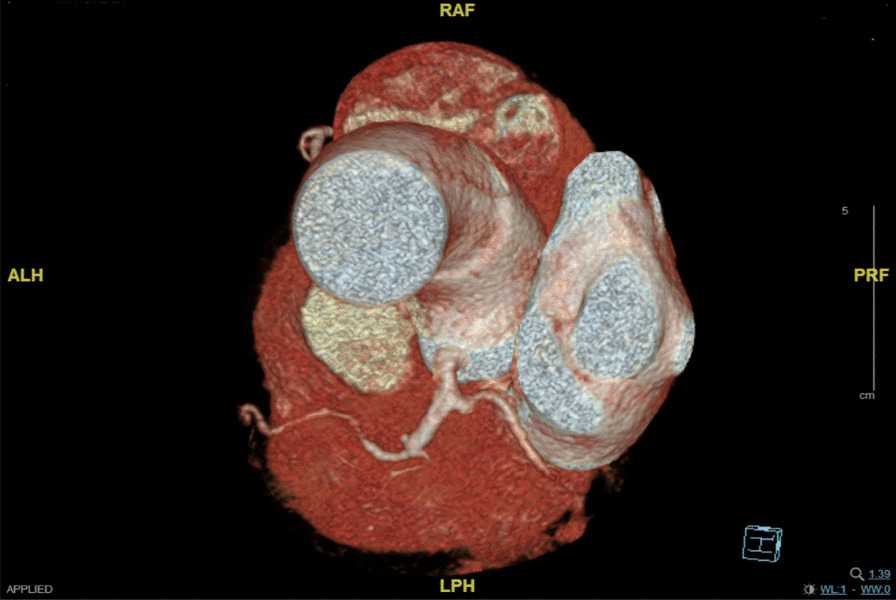
A reconstructed 3D coronary computed tomography angiogram image after the surgery showed successful correction of the lesions

Our patient was a 66-year-old male presenting with exertional substernal pain for three months. After discovering a giant angioma of 2.23 * 1.81 cm in the first diagonal artery (Fig. 1) by the local hospital on CCTA, The patient went to our tertiary medical center for further evaluation. His past medical history was positive for 1-year controlled hypertension, and he was an active smoker. On examination, his height was 178 cm, and his weight was 68 kg. His BMI was 21.46. His initial vitals included a temperature of 36.5 °C, blood pressure on the left arm of 143/90 mmHg, a regular heart rate of 61 beats per minute, and a respiration rate of 18 per minute. Physical examination revealed nothing positive. His cardiac enzymes, N-terminal pro-B-type natriuretic peptide, lipid panel, and inflammatory markers were within the normal range. The 12-lead electrocardiogram showed sinus rhythm without signs of ischemia or left ventricular hypertrophy. His chest x-ray reported a regular cardiac silhouette. Transthoracic echocardiography concorded normal cardiac morphology and function except for widened aortic sinus of 41 mm in diameter. Coronary angiography eventually identified a coronary artery to pulmonary artery fistula with giant aneurysmal dilation (Fig. 2) (Additional file 1). Multidisciplinary consultations agreed to diagnose the coronary artery to pulmonary artery fistula and giant coronary artery aneurysm (defined as diameter ≥ 2 cm) while considering surgery as a suitable treatment option. We spotted a coronary aneurysm of 3 cm in diameter located within the epicardial adipose tissue (Fig. 3). The fistula originated from the proximal left descending artery, navigated tortuously, and drained into the pulmonary artery. The surgeons opened the aneurysmal cavity and removed a 10-mm newly formed thrombus after the ligation of both the input and output vessels. They sutured the shunt, performed aneurysmorrhaphy, and excised the excess aneurysmal wall for histological analysis. The result revealed moderate intimal fibrosis, degenerated media, and decreased smooth muscle layer without evidence of inflammation, vasculitis, or atherosclerosis (Fig. 4). We concluded the final diagnosis of the congenital coronary artery to pulmonary artery fistula, giant coronary aneurysm, and concomitant thrombosis. The surgery was uneventful, and the postoperative coronary CTA confirmed the disappearance of the fistula and the aneurysm (Fig. 5). Our patient was successfully discharged without chest pain after he fully recovered from the surgery. He remained asymptomatic and emotionally satisfied at a 2-month follow-up telephone visit.

### Informed consent

We have obtained the informed consent from our patient after the admission.

## Discussion and conclusion

We report a patient diagnosed with congenital coronary artery-to-pulmonary artery fistula, giant coronary aneurysmal dilatation, and thrombosis. He received in-time surgery, and the pathology study confirmed the etiology.

CPAF, defined as the abnormal vascular communication originating from the coronary artery and draining into the pulmonary artery, accounts for 15.0–30.0% of all coronary artery fistulas (CAF), with the prevalence from 0.17 to 0.68% in the general population [3–5]. Most CPAF can be classified as the anterior type characterized by connecting the proximal portion of the left or right coronary artery and the anterior wall of the pulmonary artery. In contrast, the posterior type originates from the left circumflex artery or left sinoatrial node artery and drains into the right pulmonary artery via transverse sinus [6]. While CPAF is primarily congenital, it can also result from iatrogenic procedures, trauma, chest radiation, and several diseases like myocardial infarction, Kawasaki disease, and Takayasu arteritis [7, 8]. Patients with CPAF are usually asymptomatic due to the small size of the left-to-right shunts and carry a seemingly benign prognosis [2, 5]. Nevertheless, CPAF will cause symptoms suggestive of myocardial ischemia or even induce myocardial infarction when the shunts are large enough to create the coronary steal phenomenon [7, 9]. Large left-to-right shunts could also lead to pulmonary hypertension and induce congestive heart failure [4]. Besides, If other cardiac comorbidities such as patent ductus arteriosus, ventricular septal defect, valvular disease, and coronary atherosclerosis exist, CPAF might exacerbate the concomitant conditions [6]. Moreover, CPAF could complicate coronary aneurysmal dilatation, thrombosis, or infective endocarditis [10]. Notably, the incidence of coronary aneurysmal dilatation CPAF patients is around 15–35% [4, 6, 8]. CPAF with a ruptured coronary aneurysm carries significant mortalities through triggering medical emergencies such as cardiac tamponade or mediastinal hemorrhage [10–12].

On the aspect of diagnosing CPAF, around 47% of patients with CPAF might have continuous cardiac murmurs, best auscultated in the second intercostal space left of the sternum [7, 13]. Echocardiography could delineate CPAF’s anatomy and assess the hemodynamic changes [7]. CCTA possesses superior diagnosing capabilities owing to its non-invasiveness, relatively high spatial resolution, and 3-dimensional reconstructed imaging of the complex anatomy [4]. While coronary angiography serves as the diagnostic reference tool with its excellent visualization, it might produce false results due to the limitation of 2-dimensional projection [14]. A myocardial perfusion scan could be a valuable tool assessing hemodynamic consequences caused by CPAF [15]. Magnetic resonance imaging might also outline the conduits of CPAF [16]. Hence fully, multimodal imaging techniques are warranted for assessing and diagnosing CPAF accurately [9].

As for the treatments, surgical ligation should always be an option, especially when the patient is symptomatic, CPAF is hugely tortuous, multiple CPAFs exist, CPAF is high-flow, or CPAF combines with the giant coronary aneurysm or infective carditis [6, 17]. Endovascular embolization might also be adopted if CPAF locates proximally and consists of a single narrow drainage site without concomitant cardiac disorders requiring surgery [3, 18]. Although no consensus exists regarding the oral medication regimen, several cases utilize antiplatelet medicine, anticoagulants, beta-blockers, and calcium channel blockers to prevent thrombosis and control symptoms [2].

As for our patient, he exhibited angina pectoris in his sixties, which implicated the coronary steal phenomenon. The local hospital initially diagnosed him with the coronary aneurysm on CCTA while we modified the diagnosis to coronary-pulmonary fistula with giant coronary aneurysmal dilation through coronary angiography. He did not manifest cardiac murmurs. Having ruled out possible acquired etiologies, including vasculitis and atherosclerosis, we adopted surgery based on his persistent symptoms and concomitant large coronary aneurysm. Ultimately we performed the surgical ligation, the aneurysmorrhaphy, and the removal of the thrombus within the aneurysmal sac. The thrombus was newly formed, which could explain the adverse finding on coronary angiography 1 week ago. This unexpected finding during surgery further strengthened our decision of surgery over conservative management. Histology results of the aneurysmal wall reinforced our conviction of a congenital disorder. According to our literature search results, four cases of these three concomitant disorders have been reported. Ours is the first supported by evidence from coronary angiography, surgery, and histologic analysis. We list the invaluable experience our patient has kindly provide to us as follows: (1) integration of detailed history taking, thorough physical examination, and multimodal imaging techniques remain essential for correctly diagnosing and assessing CPAF with complex anatomy. Our patient demonstrates the need for integration because he exhibited no cardiac murmurs and presented with a false diagnosis by the local hospital. His echocardiography and chest x-ray were also negative until we conducted coronary angiography. (2) Exploring pertinent etiologies is necessary. According to other cases, Degenerated media and smooth muscle layer, suggested by the patient’s pathology report, could be attributed to the occurrence of aneurysmal dilatation [19, 20]. (3) In-time surgery poses a significant good prognosis on the patient with CPAF who meets with surgery indication. Our patient’s coronary angiography testified no signs of thrombus, while surgery 1 week later discovered new thrombus formation within the aneurysm sac. Acute embolization of the pulmonary artery or coronary artery could occur if our patient missed the surgery.

We report a rare case of congenital coronary artery-to-pulmonary artery fistula, giant coronary aneurysmal dilatation, and thrombosis. Combining these complex disorders necessitates the evaluation through multiple imaging modalities, whereas in-time surgery serves as the cornerstone for managing symptomatic patients with these combined diseases.

## Supplementary Information

**Additional file 1**. The patient’s coronary angiography. Coronary angiography identified a coronary artery to pulmonary artery fistula with giant aneurysmal dilation from left anterior oblique angulation

## Data Availability

The datasets used during the current case report are stored in our medical center’s electronic health record system. They are available from the corresponding author on reasonable request.

## Abbreviations

CPAF: coronary artery-to-pulmonary artery fistula
CAF: coronary artery fistulas
CCTA: coronary computed tomography angiography
